# Characterization of Periplasmic Protein BP26 Epitopes of *Brucella melitensis* Reacting with Murine Monoclonal and Sheep Antibodies

**DOI:** 10.1371/journal.pone.0034246

**Published:** 2012-03-23

**Authors:** Jinlang Qiu, Wenjing Wang, Jingbo Wu, Hui Zhang, Yuanzhi Wang, Jun Qiao, Chuangfu Chen, Goege F. Gao, Jean-Pierre Allain, Chengyao Li

**Affiliations:** 1 Department of Transfusion Medicine, Southern Medical University, Guangzhou, China; 2 Animal Science and Technology College, Shihezi University, Shihezi, China; 3 CAS Key Laboratory of Pathogenic Microbiology and Immunology, Institute of Microbiology, Chinese Academy of Sciences (CAS), Beijing, China; 4 Department of Haematology, University of Cambridge, Cambridge, UK; Universidad Nacional, Heredia, Costa Rica

## Abstract

More than 35,000 new cases of human brucellosis were reported in 2010 by the Chinese Center for Disease Control and Prevention. An attenuated *B. melitensis* vaccine M5-90 is currently used for vaccination of sheep and goats in China. In the study, a periplasmic protein BP26 from M5-90 was characterized for its epitope reactivity with mouse monoclonal and sheep antibodies. A total of 29 monoclonal antibodies (mAbs) against recombinant BP26 (rBP26) were produced, which were tested for reactivity with a panel of BP26 peptides, three truncated rBP26 and native BP26 containing membrane protein extracts (NMP) of *B. melitensis* M5-90 in ELISA and Western-Blot. The linear, semi-conformational and conformational epitopes from native BP26 were identified. Two linear epitopes recognized by mAbs were revealed by 28 of 16mer overlapping peptides, which were accurately mapped as the core motif of amino acid residues ^93^DRDLQTGGI^101^ (position 93 to 101) or residues ^104^QPIYVYPD^111^, respectively. The reactivity of linear epitope peptides, rBP26 and NMP was tested with 137 sheep sera by ELISAs, of which the two linear epitopes had 65–70% reactivity and NMP 90% consistent with the results of a combination of two standard serological tests. The results were helpful for evaluating the reactivity of BP26 antigen in M5-90.

## Introduction

*Brucellae* are gram-negative intracellular bacterial pathogens of both humans and animals. More than 500,000 new cases of human brucellosis are annually reported, which may greatly be underestimated according to the World Health Organization [1]. Since 1995 the incidence of human brucellosis has sharply increased in China [2]. Over 35,000 human cases were identified in 2010 by the laboratories of Chinese Center for Disease Control and Prevention (CDC) [3], and about 85% cases were caused by *B. melitensis* infected sheep or goats [2], [4]. Vaccination for animals is considered as the most efficient way to control brucellosis. An attenuated *B. melitensis* vaccine M5-90 has been mostly used for vaccination of sheep and goats in China [2], [4].

Protein BP26 is located in the periplasma of *Brucella* and has been identified as an important diagnostic antigen in brucellosis [5]–[7]. BP26 is highly conserved among *B. abortus*, *B. suis*, *B. ovis*, *B. canis*, *B. neotomae* and *B. melitensis*, and was sensitive and specific for diagnosis of *Brucella* infection in animals by enzyme immunoassays (EIAs) [6]–[10]. Excellent protective antigen and adjuvant activity were found with BP26 that induced elevated anibody and cellular responses [11]–[13]. However, the molecular feature of BP26 antigen remains unclear. To reveal the reactivity of BP26 antigen, this study extensively characterized the antibody recognitions of BP26 epitopes of *B. melitensis* M5-90 vaccine.

## Results

### Production of monoclonal antibodies to *B. melitensis* BP26

Recombinant periplasmic protein BP26 (rBP26) of *B. melitensis* M5-90 was produced by gene expression in *E. coli* and used as the immunogen for monoclonal antibody preparation. A panel of 16mer overlapping peptides, 9mer shortened peptides and mutated peptides derived from M5-90 BP26 were synthesized for mAb's recognition analysis and epitope mapping (**Table 1**). A total of 29 mAbs reactive to rBP26 of *B. melitensis* were selected from screening of hybridomas by indirect-ELISA. Of 29 clones, 18 IgG1 (k), 1 IgG2a (k), 8 IgG2b (k), 1 IgG3 (k) and 1 IgA (k) were identified (**Table 2**).

**Table 1:** Peptides derived from BP26 of *B. melitensis* M5-90.

Peptide	Sequence	Position
**Group A**		
P01	MNTRASNFLAAS	1–12
P02	SNFLAASFSTIMLVGA	6–21
P03	TIMLVGAFSLPAFAQE	15–30
P04	LPAFAQENQMTTQPAR	24–39
P05	MTTQPARIAVTGEGMM	33–48
P06	VTGEGMMTASPDMAIL	42–57
P07	SPDMAILNLSVLRQAK	51–66
P08	SVLRQAKTAREAMTAN	60–75
P09	REAMTANNEAMTKVLD	69–84
P10	AMTKVLDAMKKAGIED	78–93
P11	KKAGIEDRDLQTGGIN	87–102
P12	LQTGGINIQPIYVYPD	96–111
P13	PIYVYPDDKNNLKEPT	105–120
P14	NNLKEPTITGYSVSTS	114–129
P15	GYSVSTSLTVRVRELA	123–138
P16	VRVRELANVGKILDES	132–147
P17	GKILDESVTLGVNQGG	141–156
P18	LGVNQGGDLNLVNDNP	150–165
P19	NLVNDNPSAVINEARK	159–174
P20	VINEARKRAVANAIAK	168–183
P21	VANAIAKAKTLADAAG	177–192
P22	TLADAAGVGLGRVVEI	186–201
P23	LGRVVEISELSRPPMP	195–210
P24	LSRPPMPMPIARGQFR	204–219
P25	IARGQFRTMLAAAPDN	213–228
P26	LAAAPDNSVPIAAGEN	222–237
P27	PIAAGENSYNV	231–241
P28	GENSYNVSVNVVFEIK	235–250
P12′	NIQPIYVYPDDKNNLK	102–117
**Group B**		
P1101	GIEDRDLQT	90–98
P1102	IEDRDLQTG	91–99
P1103	EDRDLQTGG	92–100
P1104	DRDLQTGGI	93–101
P1105	RDLQTGGIN	94–102
P1106	DLQTGGINI	95–106
P1201	INIQPIYVY	101–109
P1202	NIQPIYVYP	102–110
P1203	IQPIYVYPD	103–111
P1204	QPIYVYPDD	104–112
P1205	PIYVYPDDK	105–113
P1206	IYVYPDDKN	106–114
**Group C**		
MutP11	KKAGIE**N**R**N**LQTGGIN	87–102
MutP12′	NIQPI**F**V**F**PDDKNNLK	102–117
**Group D**		
HCV NS3	SGLGLNAVAYYRGLDV	

Four groups of synthesized peptides are listed. Group A, 27 of 16mer peptides and 2 of 12 or 11mer peptides with 7mer overlapping deriving from BP26 of *B. melitensis* M5-90. Group B, 12 of 9mer peptides with 8mer overlapping shortening from P11 or P12. Group C, The mutated peptides basing on P11 and P12′, in which two site mutations are presented in underline bold letters. Group D, an HCV NS3 peptide deriving from an HCV 1b strain in the laboratory. A single letter was used for encoding amino acid (aa) sequence and aa position was numbered from BP26 protein.

**Table 2:** Classification of mAbs reactive to the epitopes of native BP26.

MAb	Isotype	Peptide-ELISA	Western-Blot	Dot-ELISA	Epitope type (aa position)
3D7	IgA (k)	+	+	−	L (93–101)
3H5	IgG2b (k)	+	+	−	L (93–101)
4D9	IgG2b (k)	+	+	+	L (93–101)
1G1	IgG1 (k)	+	+	−	L (104–111)
5A5	IgG2b (k)	+	+	+	L (104–111)
5B12	IgG1 (k)	+	+	+	L (104–111)
7C6	IgG2b (k)	+	+	−	L (104–111)
2A4	IgG1 (k)	+	+	−	L (104–111)
3H6	IgG1 (k)	+	+	+	L (104–111)
2H9	IgG1 (k)	+	+	+	L (102–117)
5F12	IgG1 (k)	+	+	+	L (102–117)
1A1	IgG1 (k)	−	+	+	SC (29–250)
1A6	IgG1 (k)	−	+	+	SC (29–250)
1B7	IgG1 (k)	−	+	−	SC (29–250)
1C11	IgG1 (k)	−	+	−	SC (48–131)
4A12	IgG2b (k)	−	+	−	SC (48–131)
4D7	IgG2b (k)	−	+	−	SC (129–250)
7A8	IgG2b (k)	−	+	−	SC (48–131)
5H3	IgG1 (k)	−	+	−	SC (48–131)
3H3	IgG2b (k)	−	−	+	C (48–131)
3C3	IgG1 (k)	−	−	+	C (48–131, 129–250)
2C12	IgG1 (k)	−	−	+	C (29–250)
3F9	IgG1 (k)	−	−	+	C (29–250)
4A2	IgG1 (k)	−	−	+	C (29–250)
4E6	IgG2a(k)	−	−	+	C (29–250)
4E12	IgG1 (k)	−	−	+	C (29–250)
4G1	IgG3 (k)	−	−	+	C (29–250)
3H2	IgG1 (k)	−	−	−	Un-classified
4E4	IgG1 (k)	−	−	−	Un-classified

MAbs were individually tested to react with the synthetic peptides by Peptide-ELISA, denatured and non-denatured native BP26 containing membrane protein extracts of M5-90 (NMP) by Western-Blot or Dot-ELISA, respectively. According to the nature of antigen, the epitopes recognized by mAbs were classified into three statuses of linear (L), semi-conformational (SC) and conformational (C) epitopes. Un-classified indicates artificial epitopes of recombinant BP26.

### Classification of epitope recognitions of monoclonal antibodies to *B. melitensis* BP26

**Figure 1:**
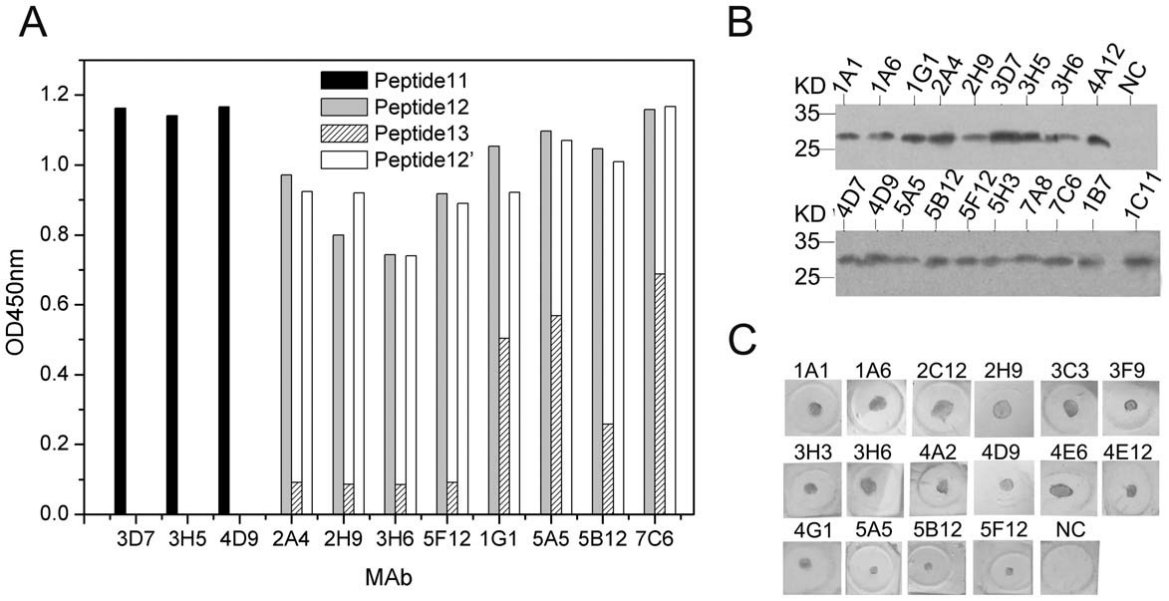
Reactivity of mAbs to 16mer peptides and native BP26 containing membrane protein extracts of *B. melitensis* M5-90. (A) Bindings of mAbs to 16mer overlapping peptides in Peptide-ELISA. Twenty nine of 16mer overlapping peptides spanning the entire sequence of BP26 were coated to the plates with 5 µg/ml in CBS buffer (pH 9.6). The peptides bound plates were tested with 29 mAb supernatants of hybridomas. An HCV NS3 peptide coated wells were used as negative-controls. Cut off was defined above 2.1 folds of OD value to negative control. The dot line indicates the level of cut off. (B) Reactivity of mAbs to the SDS denatured native BP26 of NMP in Western-Blot. (C) Reactivity of mAbs to non-denatured native BP26 of NMP in Dot-ELISA. NC, a negative control of an un-related mAb to HCV rNS3.

To classify mAb's epitope recognitions, 29 mAbs were tested for reactivity with a panel of 28 of 16mer overlapping peptides and native BP26 containing membrane protein extracts (NMP) of M5-90, respectively. Eleven mAbs reacted with three peptides P11, P12 or P13 in peptide-ELISA (**Figure 1A**), 19 mAbs reacted with the denatured native BP26 of NMP in Western-Blot (**Figure 1B**), 16 mAbs recognized non-denatured native BP26 of NMP in Dot-ELISA (**Figure 1C**), and 2 mAbs were only reactive with recombinant BP26. According to the nature of antigen, the epitopes of natural periplasmic protein BP26 were classified into three groups of linear, semi-conformational and conformational epitopes, which were recognized by 11, 8 and 8 mAbs, respectively (**Table 2**).

### Identification of BP26 epitopes recognized by monoclonal antibodies

Of 11 mAbs reacted with peptides (**Table 1** **and** **2**), mAbs 3D7, 3H5 and 4D9 were reactive with P11 (aa 87–102); mAbs 2A4, 2H9, 3H6 and 5F12 were reactive with P12 (aa 96–111); mAbs 1G1, 5A5, 5B12 and 7C6 were reactive with both P12 and P13 (aa 105–120) (**Figure 1A**). The amino acid sequences indicated that P11 and P12 spanned the different linear epitopes, while P13 shared a partly linear epitope with P12. Seven mer amino acids overlapped between P11 and P12 (**Table 1**). In order to distinguish these two epitopes, a 16mer peptide designated as P12′ (aa 102–117) within only 1mer overlapping with P11 was synthesized and showed reactivity similar to P12 in Peptide-ELISA (**Figure 1A**).

To localize the epitopes of BP26, three truncated proteins of rBP26-1 (aa 29–250), rBP26-2 (aa 48–131) and rBP26-3 (aa 129–250) were tested by ELISA for reacting with 16 semi-conformational and conformational mAbs. All of those mAbs reacted with the truncated rBP26-1 protein, 5 reacted with rBP26-2, 1 reacted with rBP26-3 and 1 reacted with both rBP26-2 and rBP26-3 (**Table 2**). Other 9 mAbs were not reactive with the shorter rBP26 proteins.

### Fine mapping for the linear epitopes of BP26

**Figure 2:**
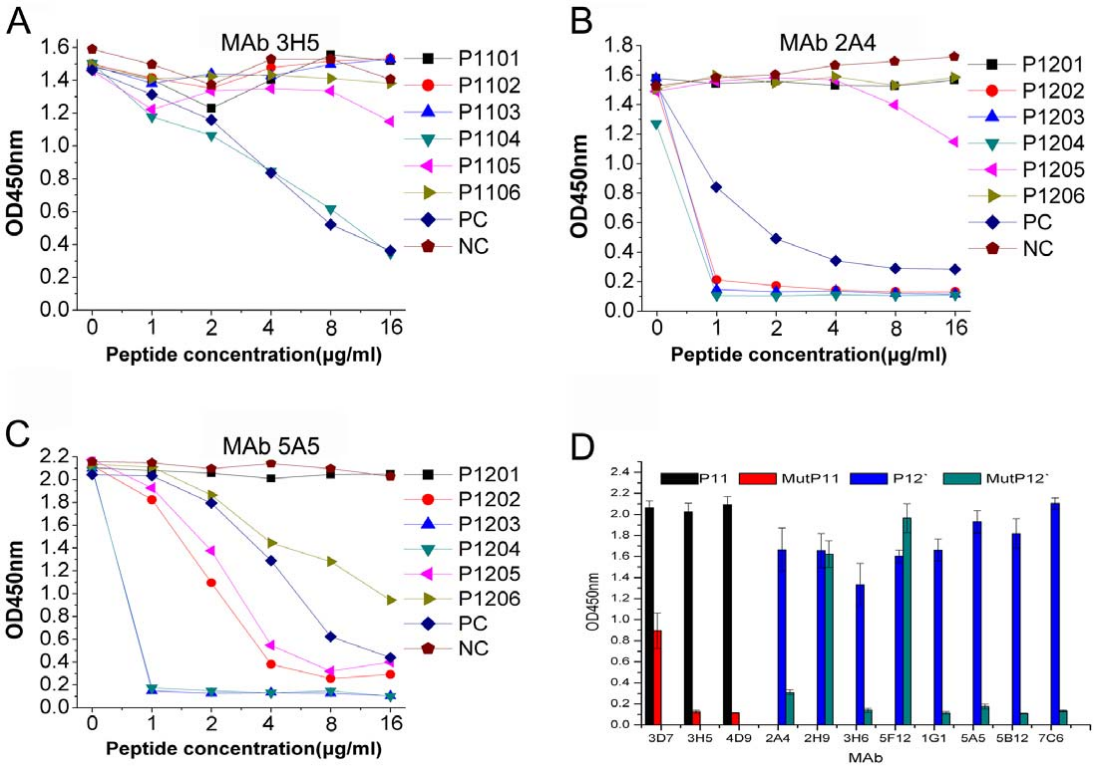
Competitive binding of mAb to the 16mer peptide inhibited by 9mer or mutated peptides in Peptide-ELISA. (A) P11 and mAb 3H5; (B) P12 and mAb 2A4; (C) P12 and mAb 5A5. A volume of 50 µl of supernatants of mAb 2A4, 3H5 or 5A5 cell cultures was pre-mixed with 50 µl of each 9mer peptide dilution (P1101 to P1106 or P1201 to P1206 and control peptides) at concentration of 0, 2, 4, 8, 16 and 32 µg/ml. A volume of 100 µl of the mAb and peptide mixture was added to the 16mer peptides P11 or P12 coated wells. P11 or P12 was used as an inhibitive (positive) control (PC), respectively. One HCV NS3 peptide was used a non-inhibitive (negative) control (NC). (D) Binding of mAbs to P11 and P12′ compared with their mutated peptides MutP11 and MutP12′.

In order to fine mapping the two linear epitopes, a panel of six 9mer peptides with 8mer amino acid overlap shortened from P11 or P12 was tested for competitive binding to mAbs 3H5, 2A4 and 5A5 in the P11 or P12 coated plates by Peptide-ELISA, respectively. The binding of mAb 3H5 to P11 was inhibited by the 9mer peptide P1104 and P11 itself (**Figure 2A**). The core motif of amino acid residues within P11 recognized by mAb 3H5 was ^93^DRDLQTGGI^101^ (position 93 to 101 within BP26). The bindings of mAbs 2A4 and 5A5 to P12 coated plates were competitively inhibited by five 9mer peptides P1202, P1203, P1204, P1205 and P1206 and P12 itself (**Figure 2B and 2C**). P1203 and P1204 strongly inhibited both 2A4 and 5A5 mAbs binding to P12, while P1202 and P1205 inhibited weakly and P1206 did not inhibit (**Figure 2B and 2C**). Comparing strongly reactive sequences of 9mer peptides and P12, the core motif of amino acid residues within P12 was ^104^QPIYVYPD^111^.

For further confirming those two linear epitopes of BP26, two mutated peptides MutP11 and MutP12′ were synthesized by substituting two amino acids D93N and D95N from P11 or substituting amino acids Y107F and Y109F from P12′, respectively (**Table 1**). The binding abilities of MutP11 and MutP12′ to 11 mAbs were compared with P11 or P12′. The reactivity of MutP11 and MutP12′ to mAbs was mostly lost or clearly decreased (**Figure 2D**), which indicated that the two mutated sites were critical to the epitopes ^93^DRDLQTGGI^101^ within P11 or ^104^QPIYVYPD^111^ within P12′ for maintaining the mAb's recognitions except for mAbs 2H9 and 5F12.

### Reactivity of linear epitope peptides and proteins of BP26 to sheep sera

**Figure 3:**
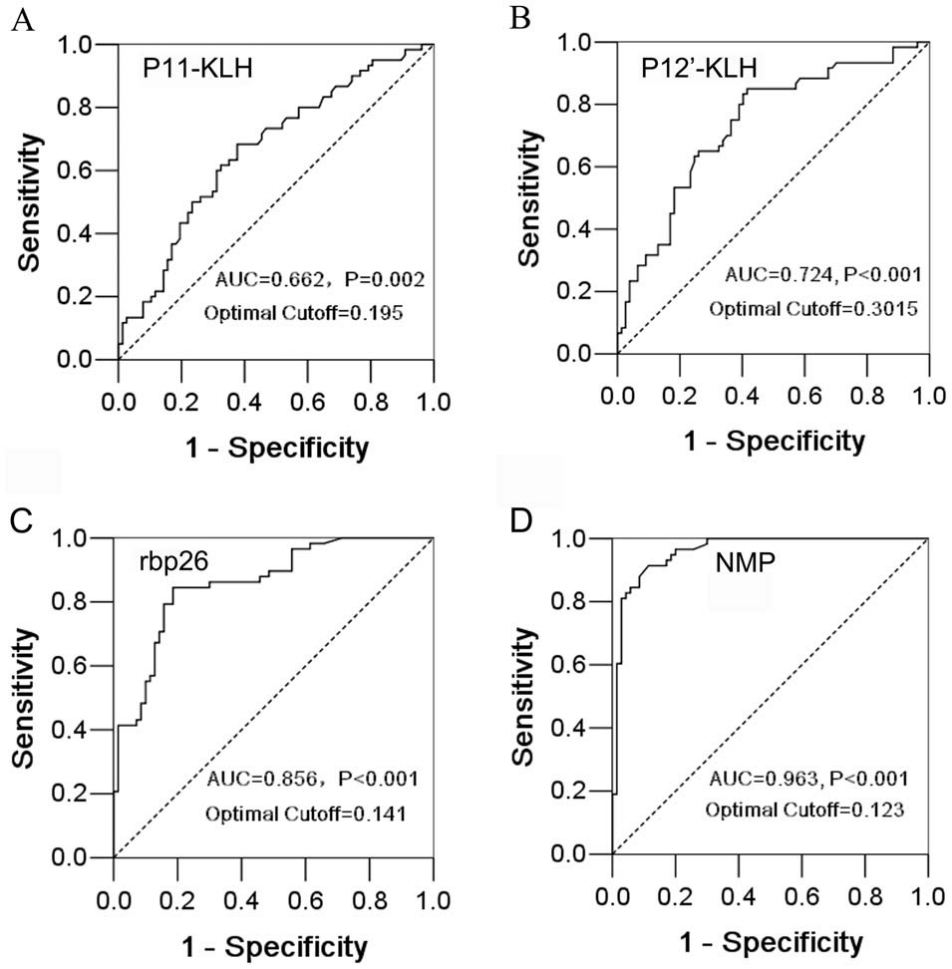
Reactivity of sheep sera to linear epitope peptides, rBP26 and NMP of *B. melitensis* in ELISAs. The receiver operating characteristic (ROC) curves were plotted using the SPSS software version 13.0. According to optimal cutoff values by the highest sum of sensitivity and specificity, the areas under ROC curves for antibody responses in sheep sera by ELISAs were compared with the results by a combination of two standard serological tests. *P* value was calculated for comparing the differences between areas under ROC curve and reference (0.5) by the Kolmogorov-Smirnov Z analysis.

To evaluate the potential capacity of those two new epitopes for raising antibody response in sheep, we tested 137 sheep sera with P11-KLH and P12′-KLH conjugates, rBP26 and native BP26 containing membrane protein extracts (NMP) of M5-90 by ELISAs. Of 137 sheep sera, 59 (39 infected and 20 vaccinated) were positive using either Standard Tube Agglutination Test (SAT) or Rose Bengal Plate Test (RBPT). Sheep antibody response to epitope peptides, rBP26 or NMP were analyzed in line with the results obtained by combination of SAT and RBPT, and their ROC curves were plotted (**Figure 3**). In term of sensitivity and specificity of peptides and proteins reacting to sheep antibodies, Figure 3 showed that the detection of reactive antibodies in the order by NMP, rBP26, P12′-KLH or P11-KLH antigens in ELISA was closer to the result by a combination of SAT and RBPT (*P*<0.001–0.002). Two linear epitope peptide conjugates (P11-KLH and P12′-KLH) were reactive to 65–70% sheep sera in peptide-ELISA consistent with the standard serological tests (*P*<0.001 and 0.002) (**Figure 3A, 3B** **and** **Table 3**), which indicated that both linear epitopes were dominant in M5-90 vaccine elicited antibodies. By using the antigens rBP26 and NMP, the ELISA had higher sensitivity and specificity for detection of antibodies to *Brucella* infection or vaccination in sheep, for which 83% or 90% were identical to the results from the combined SAT and RBPT (*P*<0.001) (**Figure 3C, 3D** **and** **Table 3**), suggesting that NMP could be a potential antigen in immunoenzyme assay (EIA) for diagnosis or quantification of antibodies to *Brucella* in infected/vaccinated animals.

**Table 3:** Evaluation for reactivity of peptides and proteins of *B. melitensis* to sheep sera in ELISAs.

Antigen	P11-KLH	P12′-KLH	rBP26	NMP
Sera dilution	1/20	1/20	1/200	1/200
Sensitivity (%)	68.3	83.3	84.5	89.7
Specificity (%)	62.3	59.7	81.4	90.0
Agreement (%)	65.0	70.1	82.8	89.8
PPV (%)	58.5	61.7	79.0	88.1
NPV (%)	71.6	82.1	86.4	91.3
True positive (a)	41	50	49	52
False positive (b)	29	31	13	7
False negative (c)	19	10	9	6
True negative (d)	48	46	57	63
Total	137	137	128	128

Reactivity of epitope peptides and proteins of *B. melitensis* to 137 sheep sera were tested, and the predictive values in ELISAs were evaluated in line with the combined SAT and RBPT. PPV, positive predictive value; NPV, negative predictive value. Sensitivity = a/(a+c); Specificity = d/(b+d); agreement = (a+d)/(a+b+c+d); PPV = a/(a+b); NPV = d/(c+d).

## Discussion

The live attenuated *Brucella melitensis* vaccine strain Rev.1 is recognized worldwide as the best vaccine available against brucellosis in sheep and goats [14]–[16]. Currently a similar attenuated *B. melitensis* vaccine strain M5-90 is mostly used in China [17], which is also considered to be an important strategy leading to the decline of brucellosis incidence in humans and animals between the 1970 and 1990s in China [2]. However, the antibody responses raised by those two live vaccines are difficult to distinguish from naturally *Brucella* infected animals using the conventional serological tests. In addition, those attenuated vaccines still keep residual virulence for humans and may result in pregnant sheep abortion [2], [18], [19]. In previous studies, a mutant obtained by deleting BP26 of Rev.1 was developed [20], which was found protective against *B. melitensis* in sheep *or B. ovis* in rams [21], [22]. In contrast, the BP26-deleted M5-90 mutant did not conserve the protective immunity from its parental vaccine strain [23], [24], which suggested that BP26 might be associated with protective efficacy in M5-90 vaccine.

Previous data demonstrated that BP26 provided a strong protection antigen within Rev. 1 inducing not only high IgG1 titers but also cellular response of IFN-γ, IL-4, IL-5 and IL-6 [11]–[13]. In a separate study, we determined the IFN-γ response specific to T cell epitopes of BP26 in sheep (**data not shown**), which also indicated that BP26 was responsible for the mixed Th cell response. The 62% (18/29) IgG1 of murine mAbs obtained in the present study, it suggested that BP26 might be associated with Th2 cytokine responses. Additionally the presence of BP26 enhanced antibody response to the chaperone protein, trigger factor (TF) [11]–[13], suggested that BP26 and other relevant proteins such as TF and omp31 might be involved in protective immunity to *Brucella* infections [25]–[28].

Molecular epitopes of BP26 to both B cells and T cells have not been explored extensively. A region of amino acid residues position 55 to 152 within a truncated BP26 protein had been previously found reactive with two monoclonal antibodies, but the epitopes recognized had not been identified [10]. In this study, 29 mAbs were produced against recombinant BP26 and 27 of them were reactive with native BP26 containing membrane protein extracts (NMP) of *B. melitensis* M5-90. Two linear epitopes were initially discovered using 28 overlapping peptides spanning the whole sequence of BP26. The core motifs of those two linear epitopes were accurately defined as amino acid sequences ^93^DRDLQTGGI^101^ and ^104^QPIYVYPD^111^ recognized by 11 of 29 mAbs, indicating that both were dominant epitopes within the BP26 antigen. Additionally the semi-conformational and conformational epitopes of native BP26 recognized by two groups of each eight mAbs were identified. All 16 mAbs were reactive to the truncated BP26-1 protein (aa 29–250) that did not contain 28 amino acid signal peptide. Four semi-conformational and one conformational epitopes were approximately localized between aa 48–131, and one semi-conformational epitope was identified between aa 129–250, which suggested that amino acids 48–131 within BP26 contained major semi-conformational and conformational epitopes. MAb 3C3 recognized both aa 48–131 and aa 129–250 of truncated rBP26 proteins, which indicated the conformational epitope was structurally formed by those two parts of BP26 protein. Other un-identified 9 mAbs might be involved in more complicated epitope recognitions, mostly conformational.

Recombinant BP26 was investigated for diagnosis of brucellosis in sheep and goats [6], [7], [10], [29], [30]. By ELISA, we compared the reactivity of sheep sera to two linear epitope peptides (P11-KLH and P12′-KLH), rBP26 and NMP in line with the combination of SAT and RBPT. Peptides P11 and P12′ reacted with sera of *Brucella* infected or M5-90 vaccinated sheep, which were 65% or 70% consistent with results of the combined SAT and RBPT. The reactivity of two linear epitopes of BP26 to mAbs could be inactivated by mutations of two critical amino acids, which suggested that molecular modification for BP26 epitopes of M5-90 could be genetically realized without altering the protein structure. Compared with rBP26, the NMP appeared more sensitive and specific in ELISA for detection of antibodies to *Brucella* from sheep, and had 90% agreement with the combination of SAT and RBPT.

In this study, we identified linear, semi-conformational and conformational B cell epitopes within *B. melitensis* BP26 and finally mapped two novel linear epitopes reacting with antibodies in sheep. Taking together with T cell epitopes of BP26, the results were helpful for evaluation of BP26 antigen function within vaccine M5-90.

## Materials and Methods

### Ethics statement

All animal care and procedures were in accordance with national and institutional policies for animal health and well-being. Mouse experimentation and Sheep blood sample collection and field study were approved by Southern Medical University (SMU) Animal Care and Use Committee (permit numbers: NFYY-2008-043 and NFYY-2010-076). All mouse surgery was performed under anesthesia, and all efforts were made to minimize suffering of animals.

### *Brucella* strain and serum

Attenuated *B. melitensis* vaccine strain M5-90 was cultured in the broth of Trypticase Soy medium (TSB) supplemented with 0.1% (w/v) yeast extract, which was used for extraction of DNA or membrane proteins. A total of 137 sheep serum samples (117 random, 20 vaccinated) were collected from the farms in *Brucella* epidemic area of Shihezi, Xinjiang or provided by the Chinese Center for Disease Control and Prevention, Beijing, China. Sera were tested by Standard Tube Agglutination Test (SAT) and Rose Bengal Plate Test (RBPT), and positive samples were confirmed by either or both tests.

### Recombinant periplasmic protein BP26

By using pET-28a plasmid, recombinant entire BP26 (rBP26), truncated rBP26-1 (aa 29–250), truncated rBP26-2 (aa 48–131) and truncated rBP26-3 (aa 129–250) were expressed in *E. coli* induced for 4 hours by 1 mM Isopropyl-1-thio-D-galactopyloranoside (IPTG) at 37°C. Soluble rBP26 was purified by Ni-NTA agarose (GE Healthcare, Milwaukee, Wisconsin, United States) and analyzed by Sodium Dodecyl Sulfate–Polyacrylamide Gel Electrophoresis (SDS-PAGE).

### Peptides and conjugates

A panel of 44 peptides was synthesized by a commercial company (Chinese Peptide Company, Hangzhou, Zhejiang, China). Twenty-eight of 16mer peptides with 7mer overlap were designated as P01 to P28 spanning the 250 amino acids of the entire BP26 periplasmic protein. Twelve of 9mer peptides shortened from P11 and P12 were designated as P1101 to 06 or P1201 to 06. One of 16mer peptide was designated as P12′, as it's only the first amino acid overlapped with the C-terminal amino acid of P11. Two mutated P11 and P12′ were designated as MutP11 and MutP12′, respectively. One HCV NS3 peptide is used as a negative control. All peptides had above 90% purity.

P11 and P12′ conjugates coupled with keyhole limpet hemocyanin (P11-KLH and P12′-KLH) were prepared by a commercial company (Chinese Peptide Company, Hangzhou, Zhejiang, China).

### *B. melitensis* M5-90 membrane protein extraction

Native BP26 containing membrane proteins (NMP) were extracted from the attenuated *B. melitensis* strain M5-90 by ReadyPrep Protein Extraction Kit (membrane II) (Bio-Rad Laboratories, Hercules, California, United States). The cells were sonicated in Lysis buffer to fractionate and isolate membrane proteins by sodium carbonate extraction methods applied in the kit [31]. The carbonate treated membranes were collected by ultracentrifugation in a Beckman Coulter 70 Ti rotor at an average of 100 000 g for 1 h at 4 C. The membrane pellet was washed and resuspended in PBS (50 mM, pH 7.2), and analyzed by SDS-PAGE.

### Mouse immunization

Three 6-weeks old BALB/c female mice were housed at the Laboratory Animal Center, Southern Hospital, Southern Medical University, Guangzhou, China. Mice were inoculated with rBP26 antigens for three times at 2-week intervals. The first subcutaneous injection contained 100 µg of rBP26 in a final volume of 100 µl (100 µg/100 µl) of complete Freund adjuvant (Sigma-Aldrich, St Louis, Missouri, United States). The second subcutaneous injection was 50 µg/100 µl rBP26 antigen with incomplete Freund adjuvant (Sigma-Aldrich, St Louis, Missouri, United States). The final boost was intraperitoneally injected with 50 µg of rBP26 in 100 µl of PBS. The antibody titers to rBP26 were determined on day 3 after the final injection, and the best responding mice were sacrificed and the spleens were obtained. Pre- and post-immunization sera were collected and used as negative or positive controls for screening monoclonal antibodies (mAbs).

### Monoclonal antibody production

The spleen cells of rBP26 immunized mice were fused with SP2/0 myeloma cells in a ratio of 5∶1 by polyethylene glycol 4000 (PEG 4000, Sigma-Aldrich, St Louis, Missouri, United States). Mouse myeloma cell line SP2/0 was provided by the laboratory, which was originated from Division of Transfusion Medicine, Department of Haematology, University of Cambridge, UK [32]. Antibody-producing hybridomas were primarily screened by an indirect ELISA with 3 µg/ml of rBP26 coated Nunc Immuno microtiter plates (JET BIOFIL Bio-filtration Products Co. Ltd, Guangzhou, Guangdong, China). Single hybridoma cell was cloned by limiting dilution in RPMI1640 (Hyclone Lab Inc, Nampa, Idaho, United States) supplemented with 20% fetal calf serum (PAA Laboratories, Tawasu, Makira-Ulawa, Solomon Islands, Australia), 100 IU/ml penicillin and 100 µg/ml streptomycin. Supernatants of cloned hybridoma cultures were collected for mAb analyses. MAb Isotyping was performed by IsoQuick Strips for Mouse Monoclonal Isotyping (Sigma-Aldrich, St Louis, Missouri, United States) or Mouse Monoclonal Antibody Isotyping Kit (Hycult Biotech Inc, Uden, Netherlands). One mAb (IgG 1 kappa) to recombinant HCV NS3 (rNS3) was used as an un-related negative control.

### Peptide-ELISA

Nunc Immuno microtiter plates were coated with 5 µg/ml of 16mer peptides in 0.1 M carbonate buffer (pH 9.6) and were incubated overnight at 4°C as described previously [32]. Plates were washed four times with phosphate-buffered saline (PBS, pH 7.2) containing 0.05% Tween-20 (PBST) and blocked in 4% bovine serum albumin (BSA) PBS buffer for 1 h at 37°C. For detecting mAb reactivity to peptides, 100 µl of hybridoma supernatant was added into each well of plates; goat anti-mouse IgG and IgM horseradish peroxidase (HRP)-conjugate (Rockland Immunochemicals Corp, Boyertown, Pennsylvania, United States) was used as secondary antibody, and 3,3′-5,5′_tetramethylbenzidine (TMB) was used as the colorimetric substrate. The reaction was determined by a microplate reader at the absorption of 450 nm (Model 550, Bio-Rad Laboratories, Hercules, California, United States).

To map epitopes accurately, a panel of 9mer shorter peptides derived from P11 or P12 was synthesized for competitive binding to mAbs in peptide-ELISA. An HCV NS3 peptide was used as negative control.

### Western-Blot

The rBP26 or native BP26 containing membrane protein extracts (NMP) of M5-90 were denatured and electrophoresed on SDS-PAGE, then transferred to Polyvinylidene Fluoride membranes (PVDF membranes, Millipore, Billerica, Massachusetts, United States), and finally blocked in TBST containing 5% non-fat dry milk for 2 h. Protein bound membranes were saturated with the diluted supernatants of mAbs cultures for 1 h at room temperature. The membranes were washed three times with TBST and incubated with 1∶10000 diluted goat anti-mouse IgG and IgM HRP-conjugate in TBST for 1 h. MAb bound membranes were washed and visualized by adding immunochemiluminescence reagent (ECL, Millipore, Billerica, Massachusetts, United States). An unrelated mAb to HCV rNS3 was used as negative control.

### Dot-ELISA

PVDF membranes (8 mm×8 mm) were soaked in methanol for 1 min and then dried at room temperature. One microliter of native BP26 containing membrane protein extract (NMP) of M5-90 was dropped onto the center of each membrane and dried at room temperature. MAbs of cloned supernatant cultures were added to NMP on the membranes and the bound mAbs were detected by goat anti-mouse HRP-conjugate. The membranes were visualized by adding diaminobenzidine (DAB) substrate for color development. An unrelated mAb to HCV rNS3 was used as negative control.

### Statistical analysis

The overall sensitivity of Peptide-ELISA, rBP26-ELISA, NMP-ELISA for detection of antibodies to *Brucella* from sheep sera were calculated using receiver operating characteristic (ROC) curves. Optimal cutoff values were defined using the highest sum of sensitivity and specificity. For each optimal cutoff value, sensitivity, specificity, positive predictive value (PPV) and negative predictive value (NPV) were calculated [33]. *P* value was obtained for comparing the differences between the areas under ROC curve (AUC) and reference (0.5) by the Kolmogorov-Smirnov Z analysis in SPSS software version 13.0.
